# Gastrointestinal Infections and Diarrheal Disease in Ghanaian Infants and Children: An Outpatient Case-Control Study

**DOI:** 10.1371/journal.pntd.0003568

**Published:** 2015-03-04

**Authors:** Ralf Krumkamp, Nimako Sarpong, Norbert Georg Schwarz, Julia Adelkofer, Wibke Loag, Daniel Eibach, Ralf Matthias Hagen, Yaw Adu-Sarkodie, Egbert Tannich, Jürgen May

**Affiliations:** 1 Bernhard Nocht Institute for Tropical Medicine (BNITM), Hamburg, Germany; 2 German Center for Infection Research (DZIF), partner site Hamburg-Borstel-Lübeck, Germany; 3 Kumasi Centre for Collaborative Research in Tropical Medicine (KCCR), Kumasi, Ghana; 4 Department of Tropical Medicine at the BNITM, German Armed Forces Hospital of Hamburg, Hamburg, Germany; 5 Kwame Nkrumah University of Science and Technology (KNUST), Kumasi, Ghana; The Hospital for Sick Children, PAKISTAN

## Abstract

**Introduction:**

Diarrheal diseases are among the most frequent causes of morbidity and mortality in children worldwide, especially in resource-poor areas. This case-control study assessed the associations between gastrointestinal infections and diarrhea in children from rural Ghana.

**Methods:**

Stool samples were collected from 548 children with diarrhea and from 686 without gastrointestinal symptoms visiting a hospital from 2007–2008. Samples were analyzed by microscopy and molecular methods.

**Results:**

The organisms most frequently detected in symptomatic cases were *Giardia lamblia*, *Shigella* spp./ enteroinvasive *Escherichia coli* (EIEC), and *Campylobacter jejuni*. Infections with rotavirus (adjusted odds ratio [aOR] = 8.4; 95% confidence interval [CI]: 4.3–16.6), *C*. *parvum/hominis* (aOR = 2.7; 95% CI: 1.4–5.2) and norovirus (aOR = 2.0; 95%CI: 1.3–3.0) showed the strongest association with diarrhea. The highest attributable fractions (AF) for diarrhea were estimated for rotavirus (AF = 14.3%; 95% CI: 10.9–17.5%), *Shigella* spp./EIEC (AF = 10.5%; 95% CI: 3.5–17.1%), and norovirus (AF = 8.2%; 95% CI 3.2–12.9%). Co-infections occurred frequently and most infections presented themselves independently of other infections. However, infections with *E*. *dispar*, *C*. *jejuni*, and norovirus were observed more often in the presence of *G*. *lamblia*.

**Conclusions:**

Diarrheal diseases in children from a rural area in sub-Saharan Africa are mainly due to infections with rotavirus, *Shigella* spp./EIEC, and norovirus. These associations are strongly age-dependent, which should be considered when diagnosing causes of diarrhea. The presented results are informative for both clinicians treating gastrointestinal infections as well as public health experts designing control programs against diarrheal diseases.

## Author Summary

Gastrointestinal infections are frequent in many low-income countries. However, their role in diarrheal diseases is still under discussion. Many epidemiological studies focus on individuals with diarrheal symptoms only, ignoring the fact that infections may progress asymptomatically as well. In order to identify infectious agents associated with diarrhea it is imperative to consider cases without symptoms as a control group. We conducted a case-control study, including 548 children with diarrhea and 651 children without gastrointestinal symptoms in order to untangle the role of gastrointestinal infections in diarrheal disease. As shown in other studies infections with rotavirus, *Shigella* spp./EIEC and norovirus are responsible for the main diarrhea burden. Co-infections are frequently observed in our study group and some organisms occur more frequently in the presence of a second one. Especially *Giardia lamblia*, which is not associated with diarrhea, is more often observed along with *Campylobacter jejuni* and norovirus, which are responsible for a high number of diarrheal episodes. This may be of particular interest since *G*. *lamblia* is, with a frequency of 40% within the study group, the most prevalent organism observed. Furthermore, the high number of co-infections challenged the identification of causative pathogens since diagnosing a particular isolate may not rule out the effect of another potentially infectious agent in diarrheal disease. We observed a strong effect of age on the course of an infection, which may guide clinicians when diagnosing causes of diarrhea.

## Introduction

Diarrheal diseases are the second leading cause of childhood mortality worldwide. In 2010, diarrhea was responsible for 0.8 million deaths of children below the age of five years, accounting for 10.5% of all deaths within that age group [1]. Mortality and morbidity patterns differ across geographical regions, with 78% of all pediatric diarrhea-associated deaths occurring in the African and South-East Asian World Health Organization (WHO) Regions [2].

The etiology of diarrhea is often not completely understood, especially in developing countries, including those in sub-Saharan Africa. Knowledge of the distribution and impact of infectious agents in diarrheal diseases is crucial in guiding empirical medical treatment and in designing prevention programs. However, many studies on the epidemiology of gastrointestinal infections are restricted to only patients with diarrhea, ignoring the possibility that infections may progress asymptomatically or even influence one another. This may be of particular importance in areas in which certain infectious agents are endemic, which would results in a high probability of ongoing infections after the development of partial immunity and/or tolerance. Thus, an adequate control group is essential to determine the pathogenicity of infectious agents, their fractions attributable to gastrointestinal symptoms (GIS), and the age-dependent association of infectious agents with GIS [3,4].

This hospital-based case-control study in a rural area of Ghana was designed to analyze gastrointestinal infections in children with and without diarrhea. The aims of this study were (i) to identify the causative pathogens linked to diarrhea, (ii) to describe their pathogenicity and contribution to the burden of diarrhea, and (iii) to analyze the frequency and interactive effects of co-infections.

## Methods

Data were collected at the Agogo Presbyterian Hospital (APH), a district hospital with approximately 250 patient beds located in the Asante Akim North municipality in Ghana. Among other facilities, it has a children’s Outpatient Department (OPD) and a pediatric ward. Asante Akim North municipal area has a population of approximately 142,400 inhabitants, spread over an area of 1,160 square kilometers. The region has a tropical climate and is mainly covered by secondary rain forest and cultivated land [5]. Falciparum malaria is highly endemic in this area [6] and HIV is, with a prevalence of 3.0% in pregnant women in 2009, at a stabile state [7].

This case-control study included children aged up to 13 years who visited APH between June 2007 and October 2008. Stool samples were collected from children with diarrhea attending the hospital’s OPD. Diarrhea was defined as at least three episodes of loose stools within the previous 24 hours. Therefore, a stool container was handed to guardians of cases and controls to collect a sample. In case a child could not provide stool at the OPD, parents were asked to return a sample to the hospital within a day after collection. Assistance was provided if needed (3). The laboratory personnel was required to confirm loose stool consistency of the collected samples. Throughout the study period, each day stool samples were also collected from children who visited the hospital OPD without diarrhea and vomiting, again with the laboratory personnel’s confirmation that stool samples were of solid consistency. Children with ongoing diarrheal disease, defined as repeated hospital visits because of diarrhea within a 6-week period, were excluded from the analysis. Data per individual were used to describe the study groups. Data per hospital visit were used for further analyses.

### Ethics statement

The Committee on Human Research, Publications and Ethics, School of Medical Science, Kwame Nkrumah University of Science and Technology, Kumasi, Ghana, approved the study design and the informed consent procedure. All participants were informed of the study’s purpose and procedures. Written informed consent was obtained from the parents or the legal guardian on behalf of the study children prior to study enrolment. Non-participation had no effect on the medical treatment provided.

### Microbiological analyses

Stool samples were refrigerated (4°C) immediately after collection and transported within a day to the laboratories at the Kumasi Centre for Collaborative Research in Tropical Medicine (KCCR). Sample transport took about 1.5 hours and a cool-box was used to maintain the cold chain. Upon arrival stool samples were aliquoted (3 x 0.2 mg) and frozen at -20°C while the reaming sample was processed for further analyses. Microscopy of blood and stool samples took place at the KCCR laboratory and DNA extractions, polymerase chain reaction (PCR) assays and ELISA-tests were conducted at the Bernhard Nocht Institute for Tropical Medicine (BNITM) in Germany. Therefore, frozen stool samples were transported on dry ice to the BNITM maintaining a temperature of -20°C (3). All samples were handled, stored and transported according to good laboratory practice.

Sodium acetate-acetic acid-formalin (SAF) solution was added to the sample to preserve parasites. Following formalin-ether concentration of protozoan cysts and trophozoites, as well as helminth eggs, fractions of the concentrated material were stained with iodine or by the modified acid-fast method, the latter to improve detection of Coccidia species, such as *Cryptosporidium*, *Cyclospora*, and *Cystoisospora* [8,9]. The samples were then viewed under a light microscope.

For molecular detection, DNA was extracted from the frozen stool samples using QIAamp DNA Stool Kits (Qiagen, Hilden, Germany). Specific sequences were amplified by PCR to identify the following organisms: *Campylobacter jejuni* [10], *Cryptosporidium parvum/hominis* [11], *Cyclospora cayetanensis* [12,13], *Entamoeba dispar* [14,15], *Entamoeba histolytica* [14,15], *Giardia lamblia* [11], norovirus [16], *Salmonella enterica* [10], *Shigella* spp./enteroinvasive *Escherichia coli* (EIEC) [10], and *Yersinia enterocolitica* [10]. Diagnostic PCR assays give the Ct value as a semi-quantitative result and a cut-off of 35 cycles was applied to determine a positive test result. As *Shigella* spp. and EIEC both possess the *ipaH* gene, these organisms cannot be distinguished by PCR [10]. Further, the PCRs applied did not allow differentiation between typhoidal and non-typhoidal Salmonella or between norovirus genotypes. Rotavirus was identified by an enzyme-linked immunosorbent assay (ELISA; Ridascreen).

Blood samples were obtained from all participants by finger prick. Thick and thin smears were prepared from these blood samples, which were stained and examined by microscopy. Malaria was defined as any asexual parasitaemia with body temperature >38°C.

All PCR reactions, as well as malaria and stool microscopy, were regularly evaluated by external and internal quality assessments. The study personal was trained to adhere to the standard operational procedures for each laboratory method.

### Statistical and epidemiological analyses

A sample size of about 500 children per group was estimated to identify isolates in 5% of the controls and 10% of the cases, considering an alpha-level of 5% and a power of 80%. Categorical variables are reported as frequencies and percentages, whereas continuous variables are reported as means ± standard deviations (SDs) or as medians with interquartile ranges (IQRs). Missing values were excluded from analyses, thus the denominators for some comparisons differ.

Because microscopy has decreased sensitivity and specificity in diagnosing diarrheal samples [8,17], all statistical comparisons were based on PCR- or ELISA-based diagnoses. The associations between diarrhea and gastrointestinal infections were determined by calculating odds ratios (OR) and 95% confidence intervals (CI). Subjects were stratified to show effects within categories of a third variable to assess and account for confounding or effect modification. Mantel-Haenszel adjusted ORs (aOR) were calculated from the stratified analyses. The attributable fractions (AF) on the diarrhea burden, defined as the proportion of diarrhea attributable to a certain pathogen, were calculated as described [18], from logistic regression estimates, including dummy variables, for age categories.

Heterogeneity in the occurrence of co-infections was assessed by comparing the probability of organism A in the presence of organism B over the probability of A in the absence of B. These associations were determined by calculating the risk ratio (RR), using the formula RR = *P*(A|B = 1)/*P*(A|B = 0), in which a value of about one indicates independence and a value different from one indicating dependence. RRs were calculated for the total study group and for cases and controls separately. Organisms diagnosed by PCR or ELISA and detected in more than 5% of stool samples in the respective study groups were included in this calculation to ensure a sufficient number of co-infections. Age-adjusted RRs (aRR) were calculated to account for the age-dependence of infections. Age was categorized into the groups 0–<1, 1–<2, 2–<5 and 5–15 years to analyze age specific infection dynamics.

All data analyses were performed with STATA 12 (StataCorp LP, College Station, USA).

## Results

In total, 1,234 patient visits made by 1,168 children were included in the analysis. The majority of children visited the hospital once (n = 1,109; 94.9%), 52 (4.5%) visited twice and 7 (0.6%) three times. Fifty-seven (4.6%) patients were admitted to the children’s ward; the other children were treated at the OPD. Girls were slightly under-represented (n = 536; 45.9%). The median age of the attendees was 33 months (IQR: 15–70 months). Stratification by age showed that, at the time of visits, 227 (18.4%) children were aged 0 to <1 year, 266 (21.6%) were aged 1 to <2 years, 375 (30.4%) were aged 2 to <5 years, and 366 (29.7%) were aged 5–13 years. Malaria was diagnosed during 236 (20.7%) visits. Differences shown in measles and yellow fever vaccination status were not observed in an age-stratified comparison.

Diarrhea was present in 548 (44.4%) cases (case visits), but absent in 686 (55.6%) instances (control visits). Fever was the most frequent disease symptom and more often observed in controls than in cases [n = 568 (82.8%) vs. n = 397 (72.5%)]. Acute malnourishment was observed in 57 (12.0%) case and 35 (9.4%) control visits., 218 (39.8%) cases suffered from vomiting. The proportion of children with diarrhea decreased gradually with age. The median ages of children with and without diarrhea were 18 months (IQR: 9–36 months) and 57 months (IQR: 26–93 months), respectively (Table 1).

**Table 1:** Characteristics of the total populations and stratified groups of case and control children.

Characteristic	Total (N = 1,234)	Cases (N = 548)	Controls (N = 686)
Sex, female (%)^#^	536 (45.9)	241 (47.6)	296 (44.6)
Age, median months (IQR)^#^	33 (15–70)	18 (9–36)	56 (25–92)
Malaria (%)^§^ ^,^ ^a^	236 (20.7)	90 (17.7)	146 (23.0)
Referred to ward (%)^§^	57 (4.6)	42 (7.7)	15 (2.2)
Mother’s age, mean years (SD)^#^ ^,^ ^b^	30.3 (7.0)	28.4 (6.2)	31.9 (7.2)
Immunisation (%)^#^			
DPT/HIB/Hep B1/Pol^c^	1,086 (93.5)	468 (93.2)	618 (93.2)
Measles^d^	925 (82.2)	370 (74.0)	583 (88.3)
Yellow fever^e^	922 (82.0)	368 (73.6)	582 (88.3)
Symptoms (%)^§^			
Fever	965 (78.2)	397 (72.5)	568 (82.8)
Acute malnourished	92 (10.8)	57 (12.0)	35 (9.4)
Vomiting	NA	218 (39.8)	NA

Abbreviations: IQR, interquartile range; SD, standard deviation; DPT/HIB/Hep B1/Pol: diphtheria, pertussis, and tetanus/Haemophilus influenzae type B/Hepatitis B/Polio; NA, not applicable.

^#^Per child (N = 1,168,)

^§^Per visit (N = 1,234)

Missing values (not considered in percentages):

^a^91,

^b^76

^c^6,

^d^8,

^e^9s,

^f^383.

Frequency (per cent) presented if not specified otherwise.

### Organisms detected in stool samples

Potentially pathogenic organisms as well as facultative and non-pathogenic parasites were detected 1,843 times in 915 (79.5%) stool samples. The most frequent infections were with *G*. *lamblia* (n = 470; 38.1%), *Shigella* spp./EIEC (n = 336; 27.2%), *C*. *jejuni* (n = 242; 19.6%), *Blastocystis hominis* (n = 144; 14.3%), and norovirus (n = 139; 11.3%). Sex-dependent differences were not observed. All parasites and protozoa, apart from *C*. *parvum/hominis*, tended to be less frequently observed in diarrheal compared to non-diarrheal samples. *Cyclospora cayetanensis*, *E*. *histolytica*, and *Yersinia* spp. were not detected in any of the stool samples tested (Table 2).

**Table 2:** Frequencies (per cent) of gastrointestinal organisms identified in stool samples sorted by species group and frequency of occurrence.

Organism	Frequency (%)	
	Cases (N = 548)	Controls (N = 651)
**Viruses**		
Norovirus^$^	91 (16.6)	48 (7.0)
Rotavirus^§^	89 (16.2)	11 (1.6)
**Bacteria**		
*Shigella* spp./EIEC^$^	167 (30.5)	169 (24.6)
*Campylobacter jejuni* ^$^	124 (22.6)	118 (17.2)
*Salmonella enterica* ^$^	33 (6.0)	27 (3.9)
*Yersinia* spp.^$^	0 (0)	0 (0)
**Protozoa**		
*Giardia lamblia* ^$^	176 (32.1)	279 (42.9)
*Blastocystis hominis* ^a^	34 (7.6)	110 (19.6)
*Entamoeba dispar* ^$^	30 (5.5)	62 (9.0)
*Entamoeba coli* ^b^	9 (2.0)	68 (12.2)
*Chilomastix mesnili* ^c^	11 (2.4)	37 (6.6)
*Cryptosporidium parvum/hominis* ^$^	45 (8.2)	14 (2.0)
*Iodamoeba buetschlii* ^c^	0 (0)	1 (0.2)
*Cystoisospora* ^d^	1 (0.3)	0 (0)
*Cyclospora cayetanensis* ^$^	0 (0)	0 (0)
*Entamoeba histolytica* ^$^	0 (0)	0 (0)
**Helminths**		
*Hymenolepis nana* ^e^	3 (0.7)	54 (9.5)
*Strongyloides stercoralis* ^c^	4 (0.9)	6 (1.1)
Hook worm^f^	2 (0.4)	4 (0.7)
*Ascaris lumbricoides* ^f^	0 (0)	1 (0.2)
Negative for all agents	101 (18.4)	152 (22.2)
Mono-infection	209 (38.1)	226 (32.9)
Mixed infection	238 (43.4)	308 (44.9)

Missing values (not considered in percentages):

^a^225;

^b^224,

^c^218,

^d^602,

^e^213,

^f^217

^$^identified via Polymerase Chain Reaction (PCR)

^§^identified via ELISA

**Fig 1:**
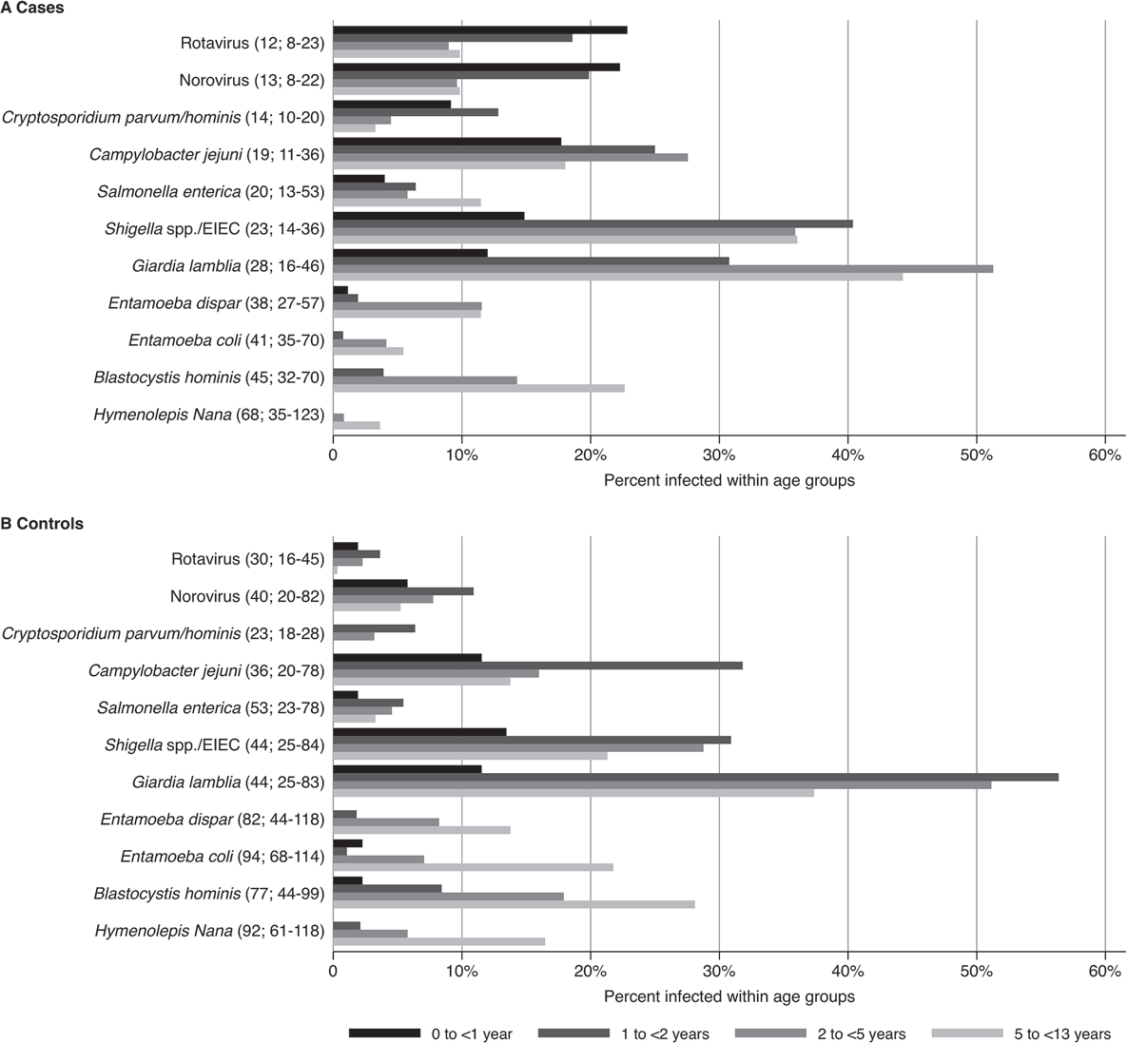
Proportions of case and control children by age group infected by various organisms. All organisms detected in more than 50 samples are shown. Median ages in months (IQR) at the time of infection are shown in brackets.

Fig 1 shows the age-stratified proportions and the median ages of case and control children infected with particular organisms. In children with diarrhea, rotavirus, norovirus, and *C*. *parvum/hominis* were most frequently observed in younger infants, with infected individuals having median ages of 12 months (IQR: 8–23 months), 13 months (IQR 8–22 months), and 14 months (IQR: 10–20 months), respectively. By contrast, control children with these organisms were older, with median ages of 30 months (IQR: 16–45 months), 40 months (IQR: 20–82 months), and 23 months (IQR: 18–28 months), respectively. For all other infections the median age in children with diarrhea ranged from 19 months (IQR: 11–36 months) to 68 months (IQR: 35–123 months). Infants below 6 months accounted for 95 (8.1%) hospital visits, with 16 stool samples (16.9%) diagnosed with rotavirus and 13 (13.7%) with norovirus. All virus-infected children, except one diagnosed with norovirus, had diarrhea. Within this age group other organisms were not detected in more than six stool samples (6.3%).

### Age-dependence of gastrointestinal infections and diarrhea

Crude analyses showed that the strongest positive associations with diarrhea were for infections with rotavirus (OR = 11.9; 95% CI: 6.2–24.9), *C*. *parvum/hominis* (OR = 4.3; 95% CI: 2.3–8.6), and norovirus (OR = 2.6; 95% CI: 1.8–3.9). Inverse associations were found for *G*. *lamblia* and *E*. *dispar* suggesting that more infections were diagnosed in controls than in cases. However, this effect was attenuated after age stratification (Table 3). Age stratification revealed varying associations of most infections with diarrhea. For example, associations with *S*. *enterica* or *Shigella spp*./EIEC increased with age, and associations with norovirus were highest in the youngest age group (OR = 4.7; 95% CI: 1.4–24.6) and lower in older children. Associations between rotavirus and diarrhea were strong amongst all age groups, although the frequency of infections decreased with age. Stratified estimates for *C*. *parvum/hominis* were lower than the crude OR, indicating that age confounded effect estimates. No associations between sex (being female) and diarrhea (OR = 0.9; 95% CI: 0.7–1.1) or rainy season and diarrhea (OR = 1.1; 95% CI: 0.9–1.4) were observed. Also, in logistic regression models these factors did not alter the association between infections and diarrheal symptoms.

**Table 3:** Crude odds ratio (OR), age-adjusted OR (aOR), and age-stratified OR on associations between gastrointestinal infections and diarrhea, sorted by aOR.

			Age stratified OR (95%-CI)			
	Crude OR (95%-CI)	aOR(95%-CI)	0 – <1 years	1 – <2 years	2 – <5 years	5–12 years
Rotavirus	11.9 (6.2–24.9)	8.4 (4.3–16.6)	15.1 (2.4–623.3)	6.1 (2.0–24.3)	8.8 (3.3–27.0)^§^	
*Cryptosporidium parvum/hominis*	4.3 (2.2–8.8)	2.7 (1.4–5.2)	2.4 (1.0–6.4)^§^		3.5 (1.1–12.2)^§^	
Norovirus	2.6 (1.8–3.9)	2.0 (1.3–3.0)	4.7 (1.4–24.6)	2.0 (0.9–4.6)	1.3 (0.6–2.8)	2.0 (0.6–5.6)
*Salmonella enterica*	1.6 (0.9–2.7)	1.7 (1.0–3.0)	1.1 (0.3–97.6)	1.2 (0.4–4.1)	1.3 (0.4–3.6)	3.8 (1.2–11.6)
*Shigella* spp./ EIEC	1.3 (1.0–1.7)	1.5 (1.1–2.0)	1.1 (0.4–3.2)	1.5 (0.9–2.6)	1.4 (0.9–2.2)	2.1 (1.1–3.9)
*Campylobacter jejuni*	1.4 (1.1–1.9)	1.3 (1.0–1.8)	1.7 (0.6–5.1)	0.7 (0.4–1.3)	2.0 (1.2–3.4)	1.4 (0.6–3.0)
*Entamoeba dispar*	0.6 (0.4–0.9)	1.2 (0.7–1.9)	1.2 (0.2–31.0)^§^		1.5 (0.7–3.1)	0.8 (0.3–2.0)
*Giardia lamblia*	0.6 (0.5–0.8)	0.8 (0.6–1.0)	1.0 (0.4–3.3)	0.3 (0.2–0.6)	1.0 (0.6–1.5)	1.3 (0.7–2.4)

Abbreviations: OR, odds ratio; CI, confidence interval; aOR, age-adjusted odds ratio.

^§^Combined age groups to avoid empty cells in cross-tabulation.

Infections manifesting at younger ages tended to be more strongly associated with diarrhea. For example, the highest ORs were observed for rotavirus, *C*. *parvum/hominis* and norovirus, infectious agents most frequently diagnosed in the younger case age groups, with median ages of 12 months (IQR: 8–23 months), 14 months (IQR: 10–20 months), and 12 months (IQR: 8–23 months), respectively. By contrast, *E*. *dispar* and *G*. *lamblia* were not positively associated with diarrhea and were more frequently diagnosed in older cases, with median ages around 38 months (IQR: 35–57 months) and 28 months (IQR: 16–46 months), respectively.

**Fig 2:**
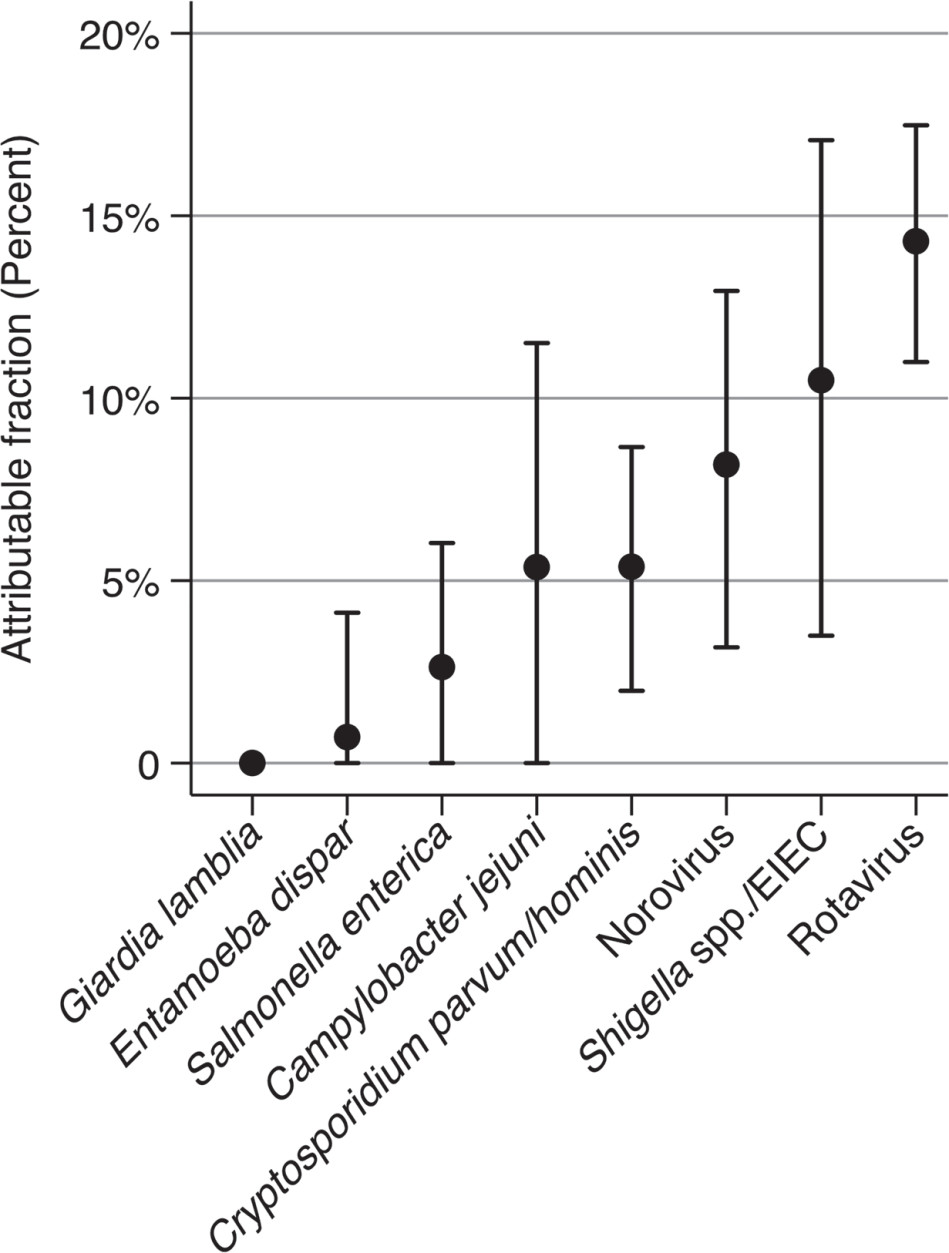
Age adjusted attributable fractions (AF) and their 95% confidence intervals of gastrointestinal infections on diarrhea.

The highest AFs (proportion of diarrheal symptoms attributable to a certain organism) were observed for rotavirus (AF = 14.3%; 95% CI: 10.9–17.5%), *Shigella* spp./EIEC (AF = 10.5%; 95% CI: 3.5–17.1%), and norovirus (AF = 8.2%; 95% CI: 3.2–12.9%), whereas all other infections had AFs of about 5% and lower (Fig 2).

### Combined infections

Probabilities for the occurrence of distinct pairs of infectious agents were calculated for organisms detected by PCR or ELISA in more than 5% of the stool samples (Table 4). Considering the total study group, most of the infections occurred independently of other organisms. However, *E*. *dispar*, *C*. *jejuni*, and norovirus were observed more often in the presence of *G*. *lamblia*, showing rate ratios of 1.6 (95% CI: 1.3–1.9), 1.3 (95% CI: 1.2–1.6), and 1.3 (95% CI: 1.1–1.6), respectively. These, estimates were comparable between cases and controls.

**Table 4:** Age-adjusted relative risk of the occurrences of selected gastrointestinal co-infections in all children and for cases and controls separately.

Combination of Organisms	aRR (95%-CI)		
	Total	Cases	Controls
*G*. *lamblia & Shigella* spp./EIEC	1.1 (0.9–1.2)	1.3 (1.0–1.6)	1.0 (0.8–1.2)
*G*. *lamblia & C*. *jejuni*	1.3 (1.2–1.6)	1.4 (1.1–1.8)	1.3 (1.0–1.6)
*G*. *lamblia &* Norovirus	1.3 (1.1–1.6)	1.4 (1.0–1.8)	1.3 (1.0–1.6)
*G*. *lamblia &* Rotavirus	0.8 (0.5–1.1)	0.8 (0.6–1.2)	NA
*G*. *lamblia & E*. *dispar*	1.6 (1.3–1.9)	1.5 (1.1–2.0)	1.6 (1.2–2.0)
*Shigella* spp./EIEC *& C*. *jejuni*	1.0 (0.8–1.2)	0.9 (0.7–1.3)	0.9 (0.7–1.3)
*Shigella* spp./EIEC *&* Norovirus	1.2 (0.9–1.6)	1.3 (0.9–1.7)	1.0 (0.6–1.6)
*Shigella* spp./EIEC *&* Rotavirus	0.8 (0.5–1.2)	0.8 (0.5–1.2)	NA
*Shigella* spp./EIEC *& E*. *dispar*	0.7 (0.4–1.1)	0.7 (0.4–1.4)	0.7 (0.4–1.2)
*C*. *jejuni & Norovirus*	0.8 (0.6–1.2)	0.7 (0.4–1.7)	1.0 (0.5–1.8)
*C*. *jejuni* & Rotavirus	0.6 (0.4–1.1)	0.6 (0.4–1.1)	NA
*C*. *jejuni* & *E*. *dispar*	1.3 (0.8–1.9)	1.4 (0.8–2.4)	1.1 (0.6–2.0)
Norovirus & Rotavirus	0.8 (0.4–1.4)	0.6 (0.3–1.1)	NA
Norovirus & *E*. *dispar*	1.0 (0.5–2.0)	1.2 (0.5–3.1)	0.8 (0.3–2.3)
Rotavirus & *E*. *dispar*	1.1 (0.4–2.9)	1.2 (0.5–3.3)	NA

Abbreviations: aRR, age-adjusted risk ratio; CI, confidence interval; NA: not applicable due to low frequency.

## Discussion

The most important cause of diarrheal disease was rotavirus, with both the highest AF and the largest risk for diarrhea across all age groups. Frequency of rotavirus infections decreased with age, but its association with diarrhea was nearly constant throughout all age groups. Similarly, a multi-center study performed in seven resource-poor countries found that rotavirus was the leading cause of diarrhea in infants, with age-dependent AFs between 16% and 28% [3]. Furthermore, 96% of children in a Mexican birth cohort were infected with rotavirus at least once by the age of 2 years. Rotavirus infections conferred protection against re-infection, resulting in less frequent and less severe manifestations in older children [19].

The proportion of norovirus infections among cases and controls was, with 16.6% and 6.8%, respectively, comparable to figures from other high-mortality developing countries, where 14% (CI: 11–16) and 7% (CI: -2–16) are reported, respectively [20]. In children with diarrhea the frequency of norovirus infections was similar to that of rotavirus. However, compared to rotavirus the amount of norovirus infections was higher in asymptomatic (control) individuals, most probably due to increasing pathogen tolerance and limited sterilizing immunity. A transmission model showed that, in highly endemic settings, protection against severe norovirus gastroenteritis could be acquired early in life, resulting in frequent asymptomatic re-infections [21]. Likewise, our study found a significant association between norovirus infection and diarrhea in infants, whereas older children were more likely to be asymptomatic carriers.

In industrialized countries, cryptosporidiosis is primarily an opportunistic infection in HIV/AIDS patients and a major cause of water-born outbreaks reported from several countries [22]. In West Africa, however, cryptosporidiosis is the cause of diarrhea in 4.9% to 14.7% of immunocompetent children, depending on age and geographical location [3,23]. Similarly, our results showed that *C*. *parvum/hominis* infections were strongly associated with diarrhea throughout all age groups and was present in more than 10% of symptomatic children below the age of 2 years. Asymptomatic cryptosporidium carriers were not observed in this age group and rarely seen in older children. A review of cryptosporidiosis in sub-Saharan Africa reported the same age distribution, with a peak amongst children aged 6–12 months. Apparently, infection can occur throughout childhood, but symptoms become less severe with age [24].

*Shigella* spp./EIEC was, after *G*. *lamblia*, the second most frequent pathogen identified in children with diarrhea, increasingly occurring in older children. However, comparing our findings with other studies is challenging because our test was PCR based. Traditionally, diagnosing shigellosis relies on culturing techniques, which selectively isolates the pathogen, followed by a biochemical identification of one of the four Shigella species [25]. Introducing PCRs techniques has their benefits but the gene sequence used for the diagnosis is also carried by enteroinvasive Escherichia coli (EIEC) [10]. Technically, our study cannot differentiate between Shigellosis and EIEC, which, in addition to the greater sensitivity of the PCR based test, might also be mirrored by the higher numbers of infections presented. Thus, despite its moderate association with diarrhea, the high prevalence of this pathogen group led to the second highest AF observed in our study.

Generally, the used PCR methods have a higher test sensitivity compared to conventional culture methods. This improves the ability to diagnose organisms in a stool sample. However, a drawback seems to be an increase in asymptomatic detections overall [26]. Since this affects diagnostics in both cases and controls the calculated ORs should not be affected. However, the estimated disease prevalence, and consequently the estimated AF, might be overestimated. Further studies using quantitative approaches [27] are needed to establish and improve diagnostic analyses for gastrointestinal diseases in low- and middle-income countries.

The burden of *G*. *lamblia* infections was quite high. Interestingly, the frequency of *G*. *lamblia* infections was lower in children with diarrhea than in asymptomatic carriers. A systematic review of the impact of *G*. *lamblia* on diarrhea highlighted that, although most studies show no or inverse effects, some studies report positive associations in children aged around 1 year, presumably as a response to initial *G*. *lamblia* infections [28]. However, the statistical power of the current study did not allow to disentangle such age-effects.

In both cases as well as controls high numbers of multiple infections were observed. Most co-infections were identified as statistically expected, although *G*. *lamblia* was more often found together with *E*. *dispar*, *C*. *jejuni*, and norovirus. *G*. *lamblia* has been reported to induce apoptosis of epithelial cells leading to increased epithelial permeability [29]. Further, *G*. *lamblia* was found to secrete proteins capable of impairing the innate immune response [30]. Alternatively, co-infections may be due to shared transmission routes. However, since most gastrointestinal organisms are transmitted via the fecal-oral route, it is unlikely that this alone explains the association among co-infections. Interestingly, a recent pooled case-control study from Ecuador also identified mechanistic interactions for diarrhea symptoms between rotavirus and *G*. *lamblia* as well as between rotavirus and *Escherichia coli* [31]. In vitro models have indicated that rotavirus may foster the adhesion, invasion, and multiplication of bacteria in enteric cells, mechanisms that may explain these synergistic effects [32–34]. Generally, the role of co-infections in diarrheal diseases deserves more attention in order to identify the associations between infections as well as interactions with GIS. Furthermore, strategies to identify causative pathogens in the presence of multiple infections are needed since diagnosing a particular isolate may not rule out other potential infectious causes in diarrheal disease.

The study presented here has several limitations, therefore the results should be interpreted with caution. Cases as well as controls were selected at a hospital OPD, thus the control group does not consist of healthy individuals. These are children seeking help for other health conditions that may increase the risk for gastro-intestinal infections. We have little background information on the total eligible study group, i.e., children that visited the OPD during the study period, from which we selected cases and controls. Thus, we cannot judge how well characteristics of cases and controls match. Table 1 highlights differences between cases and controls. For example, *falciparum* malaria is more frequently observed in controls, because controls need an alternative reason to attend the hospital, which is malaria in some attendees. Controls are also more likely to have a full vaccination schedule, which can be explained by age differences as well as due to possible differences in socio-economic status. In the analyses these factors cannot be controlled for, however, we believe that they do not act as a confounder. Even though some factors are associated with gastrointestinal infections, they are not associated with diarrheal symptoms, which would be needed to qualify as a confounder. Controls had to be diarrhea free at the point of study enrollment, yet GIS before enrollment were not assessed. Thus, controls could be carriers of pathogens if infections occurred before study enrollment. For instance, norovirus can be found in stool for up to 60 days after infection [35]. In case some controls are pathogen carriers due to recent infections study results would underestimate the actual diarrheal association. Two preconditions need to be fulfilled to generalize AFs from case-control data: (i) the case-control selection must be representative of the source population and (ii) the OR must be a robust estimator of the RR. Cases in this study were recruited from children in the OPD, making this a select group of patients seeking professional care, thereby representing individuals with moderate to severe diarrheal disease. Considering our case-control sampling approach, the OR would approximate the RR only if the rare disease assumption is fulfilled. This, however, was applicable to all infections studied, especially not for *Shigella* spp./EIEC, *C*. *jejuni*, and norovirus. In these cases, the OR is likely to overestimate the true RR, resulting in a higher AF. Several possible infectious causes of diarrhea were not detected by these methods, including adenovirus [36] enteropathogenic *Escherichia coli* [37] and enterotoxigenic *Escherichia coli* [37,38].

The AFs express the proportion of diarrheal disease that would be reduced if an organism could be removed. This measure is highly relevant to public health concerns since it demonstrates the potential effects of disease prevention and control as well as empirical disease treatment measures. In particular, it highlights the potential roles of vaccinations against rotavirus and norovirus in sub-Saharan Africa, as well as of water purification, sanitation, and hygiene measures; effective options that can reduce the burden of diarrheal diseases [39].

## Supporting Information

S1 Checklist STROBE Checklist. (DOCX)

S1 Dataset Study data. (CSV)
