# Correction: Gastrointestinal Infections and Diarrheal Disease in Ghanaian Infants and Children: An Outpatient Case-Control Study

**DOI:** 10.1371/journal.pntd.0003728

**Published:** 2015-04-22

**Authors:** Ralf Krumkamp, Nimako Sarpong, Norbert Georg Schwarz, Julia Adlkofer, Wibke Loag, Daniel Eibach, Ralf Matthias Hagen, Yaw Adu-Sarkodie, Egbert Tannich, Jürgen May

The fourth author’s name is currently spelled incorrectly. The correct spelling is: Julia Adlkofer. The correct citation is: Krumkamp R, Sarpong N, Schwarz NG, Adlkofer J, Loag W, et al. (2015) Gastrointestinal Infections and Diarrheal Disease in Ghanaian Infants and Children: An Outpatient Case-Control Study. PLoS Negl Trop Dis 9(3): e0003568. doi:10.1371/journal.pntd.0003568

In the PDF, there is an error in the placement of the heading “Organisms detected in stool samples” under the Results section. It should appear on the line below as a clear heading instead of as the last part of the previous paragraph.
