# Supplementary material for: Gastrointestinal Infections and Diarrheal Disease in Ghanaian Infants and Children: An Outpatient Case-Control Study
Source: PLoS Negl Trop Dis. 2015 Mar 4;9(3):e0003568. doi: 10.1371/journal.pntd.0003568 (PMC4349824; doi:10.1371/journal.pntd.0003568)
Supplement: S1 Checklist — (DOCX) [file pntd.0003568.s001.docx]

STROBE Statement—checklist of items that should be included in reports of observational studies

|  | | Item No. | | Recommendation | Line  No. | Relevant text from manuscript | |
| --- | --- | --- | --- | --- | --- | --- | --- |
| **Title and abstract** | | 1 | | (*a*) Indicate the study’s design with a commonly used term in the title or the abstract | 46 | This case-control study assessed the associations … | |
|  |  |  |  | (*b*) Provide in the abstract an informative and balanced summary of what was done and what was found | 49  52 | See methods  See results | |
| Introduction | | | | | |  | |
| Background/rationale | | 2 | | Explain the scientific background and rationale for the investigation being reported | 134 | The etiology of diarrhea is often not completely understood, especially in developing countries, including those in sub-Saharan Africa. Knowledge of the distribution and impact of infectious agents in diarrheal diseases is crucial in guiding empirical medical treatment and in designing prevention programs. | |
| Objectives | | 3 | | State specific objectives, including any prespecified hypotheses | 146 | The aims of this study were (i) to identify the causative pathogens linked to diarrhea, (ii) to describe their pathogenicity and contribution to the burden of diarrhea, and (iii) to analyze the frequency and interactive effects of co-infections. | |
| Methods | | | | | |  | |
| Study design | | 4 | | Present key elements of study design early in the paper | 164 | This case-control study included children aged up to 13 years who visited APH between June 2007 and October 2008 … | |
| Setting | | 5 | | Describe the setting, locations, and relevant dates, including periods of recruitment, exposure, follow-up, and data collection | 156  164 | Data were collected at the Agogo Presbyterian Hospital (APH), a district hospital with approximately 250 patient beds located in the Asante Akim North municipality in Ghana ...  This case-control study included children aged up to 13 years who visited APH between June 2007 and October 2008. | |
| Participants | | 6 | | (*a*) *Cohort study*—Give the eligibility criteria, and the sources and methods of selection of participants. Describe methods of follow-up  *Case-control study*—Give the eligibility criteria, and the sources and methods of case ascertainment and control selection. Give the rationale for the choice of cases and controls  *Cross-sectional study*—Give the eligibility criteria, and the sources and methods of selection of participants | 164 | Stool samples were collected from children with diarrhea attending the hospital’s OPD. Diarrhea was defined as at least three episodes of loose stools within the previous 24 hours. The laboratory personnel was required to confirm loose stool consistency of the collected samples. Throughout the study period, each day stool samples were also collected from children who visited the hospital OPD without any GIS, defined as the lack of diarrhea, vomiting, or acute malnourishment, again with the laboratory personnel’s confirmation that stool samples were of solid consistency. Children with acute malnourishment were ineligible to serve as controls because of reported associations with selected gastrointestinal infections [[8](#_ENREF_8),[9](#_ENREF_9)]. | |
|  |  |  |  | (*b*) *Cohort study*—For matched studies, give matching criteria and number of exposed and unexposed  *Case-control study*—For matched studies, give matching criteria and the number of controls per case | NA |  | |
| Variables | | 7 | | Clearly define all outcomes, exposures, predictors, potential confounders, and effect modifiers. Give diagnostic criteria, if applicable | 246  456 | Age-adjusted RRs (aRR) were calculated to account for the age-dependence of infections.  Table 1 highlights differences between cases and controls. For example, *falciparum* malaria is more frequently observed in controls, because controls need an alternative reason to attend the hospital, which is malaria in some attendees. Controls are also more likely to have a full vaccination schedule, which is presumably due to differences in socio-economic status. In the analyses these factors cannot be controlled for however, we believe that they do not act as a confounder. Even though some factors are associated with gastrointestinal infections, they are not associated with diarrheal symptoms, which would be needed to qualify as a confounder. | |
| Data sources/ measurement | | 8* | | For each variable of interest, give sources of data and details of methods of assessment (measurement). Describe comparability of assessment methods if there is more than one group | 189 | See “Microbiological analyses” | |
| Bias | | 9 | | Describe any efforts to address potential sources of bias | 230 | Because microscopy has decreased sensitivity and specificity in diagnosing diarrheal samples [[10](#_ENREF_10),[19](#_ENREF_19)], all statistical comparisons were based on PCR- or ELISA-based diagnoses. | |
| Study size | | 10 | | Explain how the study size was arrived at | 224 | A sample size of about 500 children per group was estimated to identify isolates in 5% of the controls and 10% of the cases, considering an alpha-level of 5% and a power of 80%. | |
| Quantitative variables | 11 | | Explain how quantitative variables were handled in the analyses. If applicable, describe which groupings were chosen and why | | 246 | Age was categorized into the groups 0–<1, 1–<2, 2–<5 and 5–15 years to study age specific infection dynamics. | |
| Statistical methods | 12 | | (*a*) Describe all statistical methods, including those used to control for confounding | | 232 | The associations between diarrhea and gastrointestinal infections were determined by calculating odds ratios (OR) and 95% confidence intervals (CI). Subjects were stratified to show effects within categories of a third variable to assess and account for confounding or effect modification. Mantel-Haenszel adjusted ORs (aOR) were calculated from the stratified analyses. The attributable fractions (AF) on the diarrhea burden, defined as the proportion of diarrhea attributable to a certain pathogen, were calculated as described [[20](#_ENREF_20)], from logistic regression estimates, including dummy variables, for age categories. | |
|  |  |  | (*b*) Describe any methods used to examine subgroups and interactions | | 233 | Subjects were stratified to show effects within categories of a third variable to assess and account for confounding or effect modification. Mantel-Haenszel adjusted ORs (aOR) were calculated from the stratified analyses. | |
|  |  |  | (*c*) Explain how missing data were addressed | | 228 | Missing values were excluded from analyses, thus the denominators for some comparisons differ. | |
|  |  |  | (*d*) *Cohort study*—If applicable, explain how loss to follow-up was addressed  *Case-control study*—If applicable, explain how matching of cases and controls was addressed  *Cross-sectional study*—If applicable, describe analytical methods taking account of sampling strategy | | NA |  | |
|  |  |  | (*e*) Describe any sensitivity analyses | | NA |  | |
| Results | | | | | | |  |
| Participants | 13* | | (a) Report numbers of individuals at each stage of study—eg numbers potentially eligible, examined for eligibility, confirmed eligible, included in the study, completing follow-up, and analysed | | NA | In our study setting the number of probable cannot be assessed. | |
|  |  |  | (b) Give reasons for non-participation at each stage | | NA |  | |
|  |  |  | (c) Consider use of a flow diagram | | NA |  | |
| Descriptive data | 14* | | (a) Give characteristics of study participants (eg demographic, clinical, social) and information on exposures and potential confounders | | 250  643 | In total, 1,199 patient visits made by 1,136 children were included in the analysis. The majority of children visited the hospital once (n=1,079; 95.0%), 51 (4.5%) visited twice and 6 (0.5%) three times. Fifty-four (4.5%) patients were admitted to the children’s ward; the other children were treated at the OPD …  Table 1 | |
|  |  |  | (b) Indicate number of participants with missing data for each variable of interest | | 643 +  658 | Table 1  Table 2 | |
|  |  |  | (c) *Cohort study*—Summarise follow-up time (eg, average and total amount) | | NA |  | |
| Outcome data | 15* | | *Cohort study*—Report numbers of outcome events or summary measures over time | |  |  | |
|  |  |  | *Case-control study—*Report numbers in each exposure category, or summary measures of exposure | | 228  658 | Potentially pathogenic organisms as well as facultative and non-pathogenic parasites were detected 1,793 times in 954 (79.6%) stool samples. The most frequent infections were with *G. lamblia* (n=455; 40.0%), *Shigella* spp./EIEC (n=326; 27.2%), *C. jejuni* (n=237; 19.8%), *Blastocystis hominis* (n=141; 14.4%) …  Table 2 | |
|  |  |  | *Cross-sectional study—*Report numbers of outcome events or summary measures | | NA |  | |
| Main results | 16 | | (*a*) Give unadjusted estimates and, if applicable, confounder-adjusted estimates and their precision (eg, 95% confidence interval). Make clear which confounders were adjusted for and why they were included | | 309 | Crude analyses showed that the strongest positive associations with diarrhea were for infections with rotavirus (OR=13.8; 95% CI: 6.9–31.5), *C. parvum/hominis* (OR=4.4; 95% CI: 2.3–9.0), and norovirus (OR=2.7; 95% CI: 1.9–4.1). Inverse associations were found for *G. lamblia* and *E. dispar* suggesting … | |
|  |  |  | (*b*) Report category boundaries when continuous variables were categorized | | 226 | Categorical variables are reported as frequencies and percentages, whereas continuous variables are reported as means ± standard deviations (SDs) or as medians with interquartile ranges (IQRs). Missing values were excluded from analyses, thus the denominators for some comparisons differ. | |
|  |  |  | (*c*) If relevant, consider translating estimates of relative risk into absolute risk for a meaningful time period | | 236 | (Attributable fractions used)The attributable fractions (AF) on the diarrhea burden, defined as the proportion of diarrhea attributable to a certain pathogen, were calculated as described [[20](#_ENREF_20)], from logistic regression estimates, including dummy variables, for age categories | |

| Other analyses | 17 | Report other analyses done—eg analyses of subgroups and interactions, and sensitivity analyses | 322  658 | However, this effect was attenuated after age stratification (Table 3). Age stratification revealed varying associations of most infections with diarrhea. For example, associations with *S. enterica* or *Shigella spp.*/EIEC increased with age, and associations with norovirus were highest in the youngest age group (OR=6.5; 95% CI: 1.6–57.0) and lower in older children …  Table 3 |
| --- | --- | --- | --- | --- |
| Discussion | | | | |
| Key results | 18 | Summarise key results with reference to study objectives | 356 | The most important cause of diarrheal disease was rotavirus, with both the highest AF and the largest risk for diarrhea across all age groups. Frequency of rotavirus infections decreased with age, but its association with diarrhea was nearly constant in all age groups. |
| Limitations | 19 | Discuss limitations of the study, taking into account sources of potential bias or imprecision. Discuss both direction and magnitude of any potential bias | 444 | The study presented here has several limitations. Cases as well as controls were selected at a hospital OPD, thus the control group does not consist of healthy individuals. These are children seeking help for other health conditions that may increase the risk for gastro-intestinal infections … |
| Interpretation | 20 | Give a cautious overall interpretation of results considering objectives, limitations, multiplicity of analyses, results from similar studies, and other relevant evidence | 365–443 | Relevant organisms are discussed with reference to the literature. |
| Generalisability | 21 | Discuss the generalisability (external validity) of the study results | 444 | Generalisability discussed in the limitations chapter |
| Other information | |  | | |
| Funding | 22 | Give the source of funding and the role of the funders for the present study and, if applicable, for the original study on which the present article is based |  | Funding statement submitted |

*Give information separately for cases and controls in case-control studies and, if applicable, for exposed and unexposed groups in cohort and cross-sectional studies.
